# Public perception of NHS general practice during the first six months of the COVID-19 pandemic in England

**DOI:** 10.12688/f1000research.52392.1

**Published:** 2021-04-08

**Authors:** Lorna J. Duncan, Kelly F.D. Cheng

**Affiliations:** 1 Centre for Academic Primary Care, Population Health Sciences, Bristol Medical School, University of Bristol, Bristol, UK; 2 Bristol Medical School, University of Bristol, Bristol, UK

**Keywords:** COVID-19, general practice, coronavirus, SARS-CoV-2 transmission, delivery model, face-to-face consultation, patient communication, patient experience

## Abstract

**Background: **In March 2020, the delivery of NHS general practice consultations was rapidly modified to mitigate against the spread of COVID-19. Remote triage and consultations became the default, with adapted models for face-to-face contact if clinically required. This study aimed to gain insight into public perception of these adaptations.

**Methods: **Two online surveys were developed, and conducted between August and September 2020. Survey A, open to anyone receiving the link to it, considered respondents’ experiences of healthcare contacts since March 2020, and their understanding of the adapted delivery. Survey B, open to survey A respondents only, then considered how healthcare communication had been received and individual preferences for this. Survey participation was voluntary.

**Results: **The views and experiences of 150 members of the public were obtained. 105 had considered contacting general practice, although half avoided this or delayed doing so for longer than usual. While some patients did so ‘to help the NHS’, others experienced reduced access for a variety of reasons including COVID-19 safety concerns. Some however reported benefitting from remote consultation availability and regular texts/emails from their practice.

68% (102/150) of respondents were unaware that patients with COVID-19 were seen separately from other patients during general practice appointments. 27% of those in survey B who had avoided or delayed contact said they would have felt more comfortable contacting general practice had they known about this.

**Conclusions: **Experience and use of the adapted general practice models varied. Some patients felt their access to healthcare was reduced, often due to technological requirements. For some who found attending face-to-face appointments difficult however, remote contact was advantageous. Most patients surveyed were unaware of the COVID-19 control measures in place during face-to-face general practice consultations. Assessment of adapted delivery model accessibility and clearer public messaging about the changes may help reduce inequalities.

## Introduction

In March 2020, with the onset of the COVID-19 pandemic, the delivery of general practice consultations changed rapidly and extensively throughout England. National Health Service (NHS) standard operating procedure was adapted to ensure the physical separation of patients with suspected or diagnosed COVID-19 (‘COVID-19 patients’) from others, to minimise cross-infection. ^1^ Remote triage and consultation became the default, with face-to-face contact only used when clinically necessary. ^1^ As a result, the proportion of face-to-face general practice consultations dropped from 80% before March 2020, to 47% in April 2020. While this rose gradually after the end of the first national lockdown in July, it remained considerably below pre-pandemic levels at 56% in the most recently available (January 2021) data. ^2^

**Figure 1.**
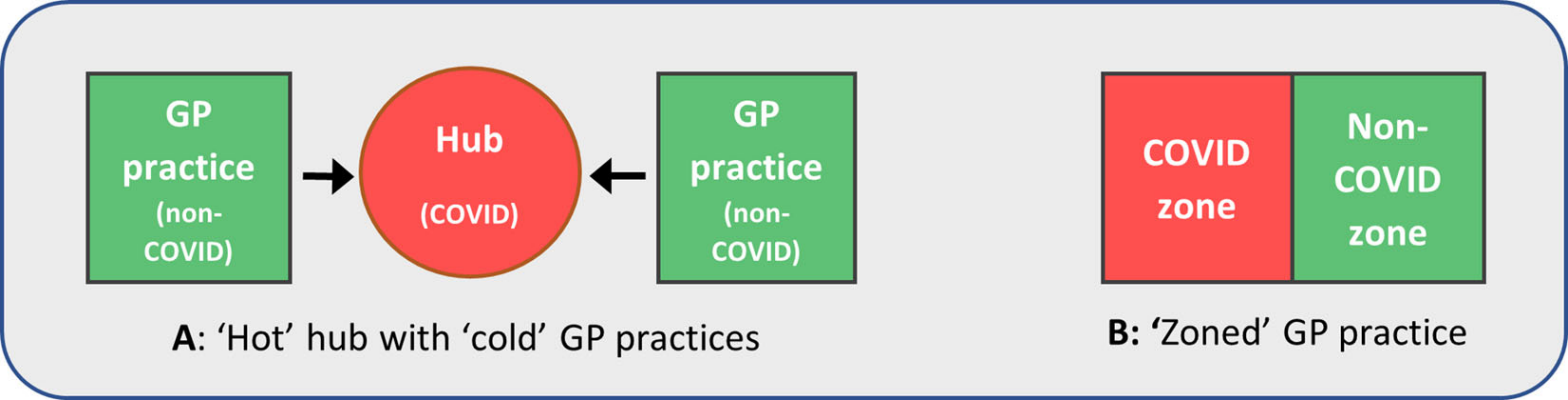
Typical models used to separate patients with suspected or diagnosed COVID-19 from others in general practice. Variations of these models may be used, as well as designated ‘COVID-19’ and ‘other’ home visiting services.

When face-to-face consultations were necessary, they required reorganisation to comply with Infection Prevention and Control (IPC) guidance. ^1^ Our June 2020 study reports on the adapted models used to deliver NHS face-to-face general practice consultations in England. ^3^ While several nuances to these models exist, the two most typical are shown in Figure 1. In model A, COVID-19 patients are seen at a ‘hot’ hub - a site shared between several locally collaborating practices. All other patients are seen at ‘cold’ GP practices. In model B meanwhile, COVID-19 and other patients are seen at their own practice, but in two separate ‘zones’. These are carefully managed to minimise cross-contamination, with staff working in one zone only, and separate entrances and exits.

In addition to the reduced proportion of face-to-face consultations, the number of all-mode general practice consultations (including telephone, video/online and face-to-face appointments as well as home visits) also dropped by around 30% in April 2020. ^2^ Among many possible reasons for this are the change to total triage prior to arranging consultations, and public response to the adapted consultation models themselves.

The aims of this study were:

- to explore public experiences and perceptions of general practice in the first 6 months of the pandemic (March-Sept 2020);
- to understand public awareness of the changes to general practice and the ways in which information had been received about this.

## Methods

Two online surveys, A and B, were conducted sequentially to identify the public’s experience and perceptions of general practice in England from March to September 2020.

### Survey design

Survey A considered:

• respondents' contacts with primary care for any symptoms since March 2020, their experience and satisfaction with this;
• respondents' awareness of the separation of COVID-19 patients from others during general practice face-to-face consultations.

Survey B then considered:

• how respondents who knew that COVID-19 patients were separated from others during face-to-face general practice consultations had received this knowledge;
• COVID-19 information sources used and preferred by respondents.

Survey questions were developed by the study team and made available using JISC online surveys. They were pre-tested on five people (two experienced in survey design), and minor changes to wording were made for clarity. The final questionnaires and flyer giving password access to survey A are available as *Extended data.* ^21^^-^^23^

Participation in both surveys was voluntary and anonymity was assured – completion of a survey indicated consent to participate. Ethical approval was not required due to the low risk nature of the surveys. Survey A was open to anybody receiving details of it, including the password. Survey B was open to survey A respondents who agreed to help further.

### Data collection

Survey A details were distributed by the Patient and Public Involvement and Engagement team (Centre for Academic Primary Care, University of Bristol) to their contacts list via email, attaching our flyer. A newsflash was also placed in People in Health West of England’s newsletter. Details were further distributed by Dr L. Farbus and others at NHS England and NHS Improvement, and by South Gloucestershire Council. Survey B was distributed from the survey website to email addresses supplied by survey A respondents.

Survey A was open August 4 ^th^—September 9 ^th^ 2020; survey B between August 19 ^th^ and September 14 ^th^ 2020.

### Analysis

Statistical analyses (counts and percentages) of closed questions were provided within the JISC online survey analysis tool. Free-text responses were analysed both numerically (grouped in relation to the items raised) and narratively by the team. Quotations in the results section represent key themes.

Additional reporting of methodology, following guidance for online surveys, ^4^ is available as *Extended data.* ^24^

## Results

### Survey A respondents

**Figure 2.**
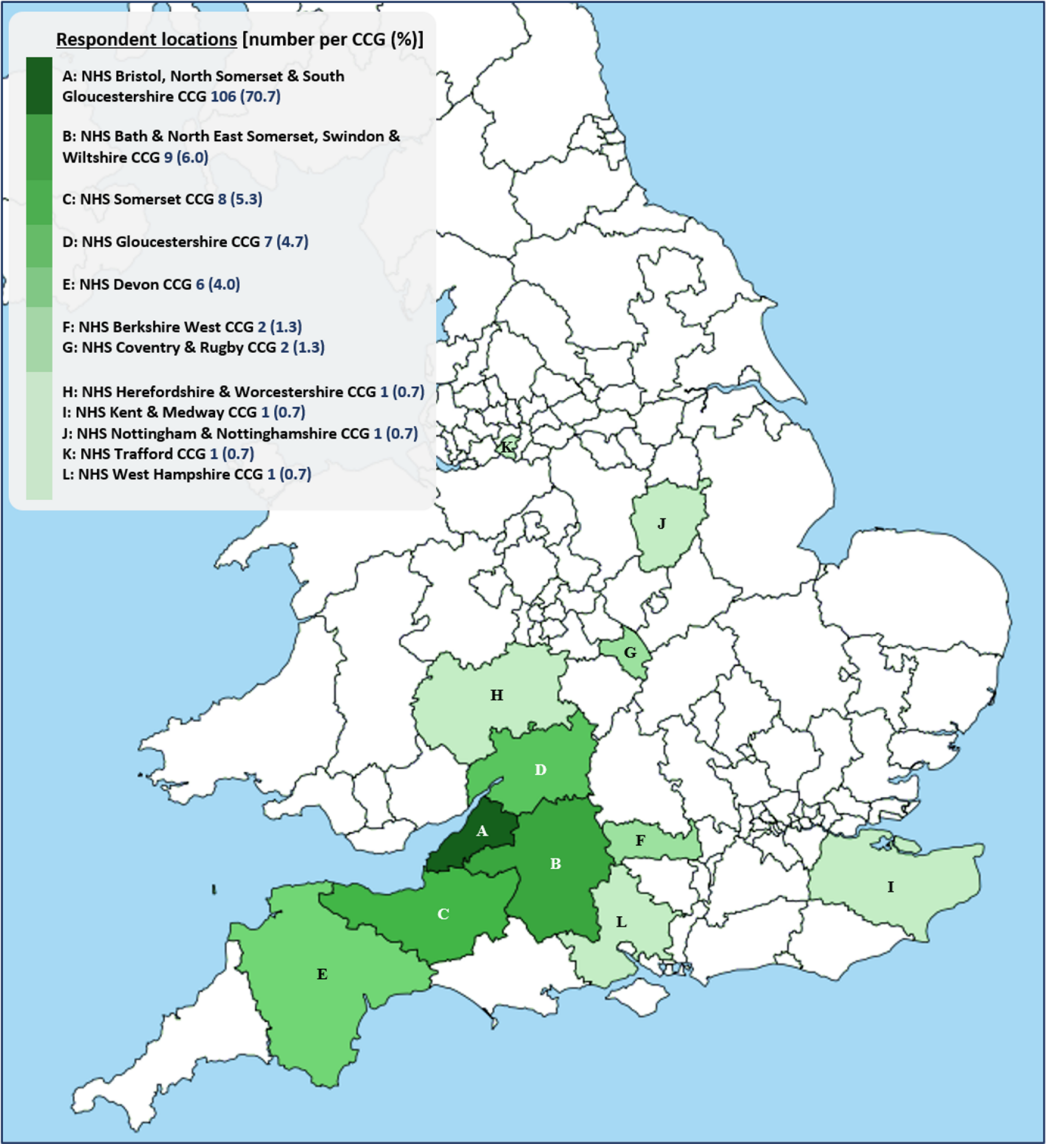
Locations of survey A respondents (n=150) by Clinical Commissioning Group (CCG). 5 respondent locations unknown.

A total of 150 people completed survey A. Figure 2 shows their locations in relation to Clinical Commissioning Groups (CCGs), organisations responsible for planning and commissioning most hospital and community NHS services in England. Our respondents lived within the boundaries of 12 CCGs, labelled A-L on the map. 91% of respondents lived in South-West England (CCGs A-E), a region of relatively low COVID-19 incidence to date. ^5^ 71% were from NHS Bristol, North Somerset and South Gloucestershire CCG. 15 respondents were healthcare professionals, with three working in general practice. Closed responses to survey A questions are available as *Underlying data*. ^20^

*Decision to contact general practice or NHS 111*

**Figure 3.**
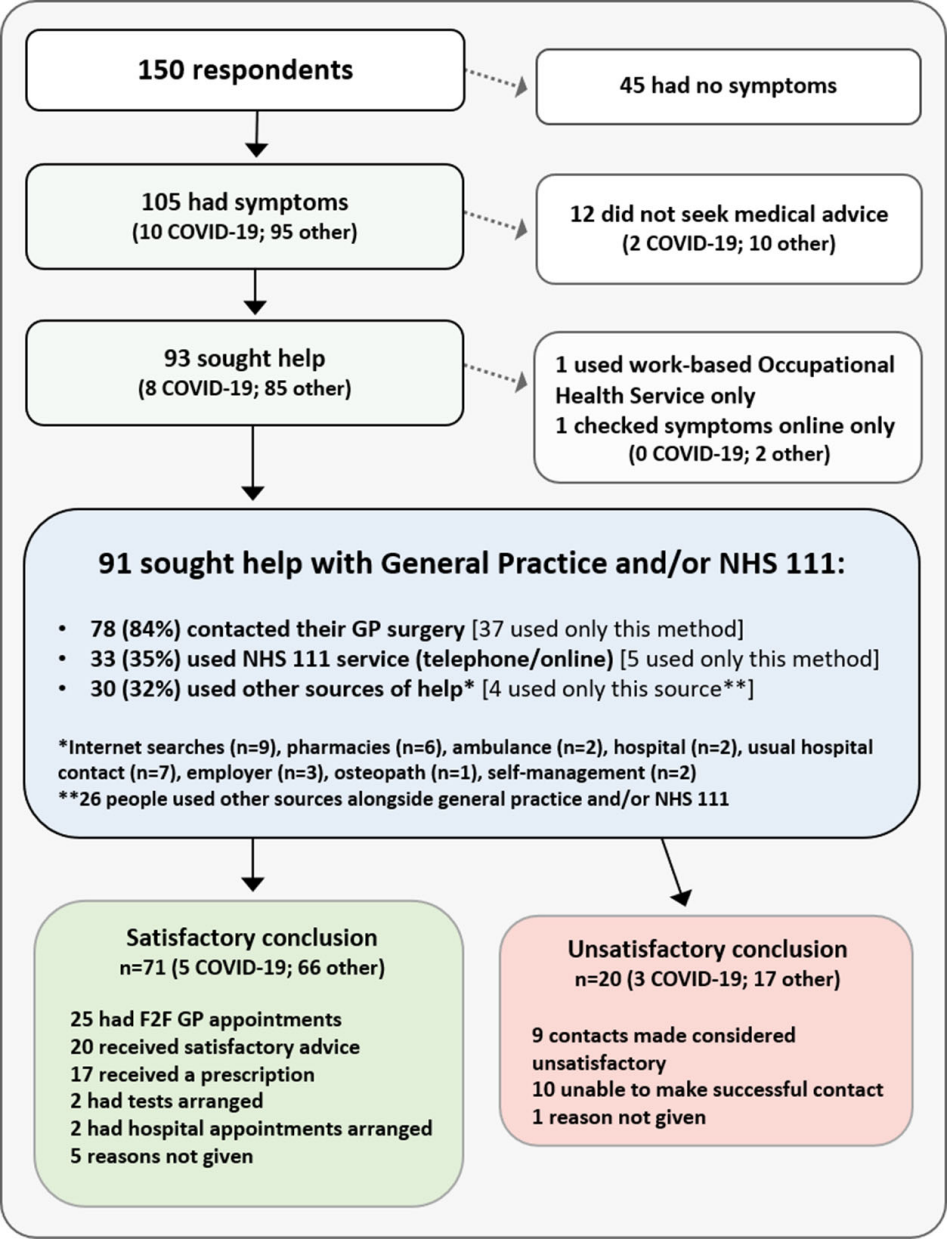
Survey A respondents’ use of healthcare, March-Sept 2020. NHS 111 is a national telephone helpline and website for patients.

In total, 70% (105/150) of survey A respondents reported having considered contacting general practice or NHS 111 (a national telephone helpline and website) since March 2020; 10 thought they may have had COVID-19. Figure 3 shows the healthcare interactions of all respondents between March and September. It can be seen that twelve symptomatic respondents did not seek advice; a further 41 reported delaying doing so for longer than usual (data not shown). Excepting four people who managed their own symptoms, these two groups represented 47% of symptomatic respondents. The most common reason for this, given by 39% of these respondents, was a desire to ‘help the NHS’. Other factors included access issues (anticipated or experienced), lack of face-to-face consultations or feeling uncomfortable with telephone consultations, and fear of contracting COVID-19.

By contrast, 11% of symptomatic respondents reported contacting general practice more quickly than usual, mainly due to symptom severity or anxiety. In two cases however, patients required prompt advice to establish whether they should self-isolate, according to COVID-19 regulations.

*Contacts made*

Irrespective of time taken to seek help, a total of 93 people - 89% of those who were symptomatic - did so. While two people used occupational health or online searches only, the remaining 91 used NHS healthcare, as detailed in Figure 3. The vast majority - 78 people - contacted their GP surgery, 37 of these using only this method. 33 respondents used the NHS 111 service (15 by telephone, 18 accessing it online), but only 5 used it alone. Several other sources (identified in Figure 3) were also used by 30 people, but most used these alongside general practice or NHS 111 contacts.

*Satisfaction with contacts*

As Figure 3 illustrates, 78% (71/91) of respondents who contacted general practice and/or NHS 111 felt they received the help they needed. This included all 25 who had face-to-face appointments, despite one-fifth having had initial concerns about attending related to COVID-19 safety. 20 respondents however were dissatisfied, with reasons involving the inability to make successful contact, or unsatisfactory outcomes where contact was made, due mainly to the unavailability of appointments and dissatisfaction with remote appointments.

*Understanding of adapted delivery models*

The evident reluctance to seek help by half of the patients in this survey was explored by investigating all 150 respondents’ understanding of the changed general practice delivery models. A large proportion (68%) reported not knowing whether COVID-19 patients were separated from others in face-to-face consultations. Similar proportions were seen in each sub-group in Figure 3. Further confusion is indicated by the fact that 25% of those who had had symptoms were unaware that face-to-face general practice consultations could happen during lockdown.

### Survey B respondents

Survey B was distributed to 71 survey A respondents who indicated their willingness to help further, and 56 of these (79%) completed it. Their characteristics were checked using responses to the first survey and this sub-group was found to be similar in terms of location (96% lived in South-West England compared to 91% in survey A) and occurrence of symptoms (73% compared to 70%). However, awareness of the separation of COVID-19 patients from others was somewhat higher in these respondents, at 39% compared to 32% in survey A respondents.

*Communication regarding COVID-19 and changes to general practice service delivery*

In total, 28% of respondents who knew about the measures used to control COVID-19 during face-to-face consultations had working links to general practice. Others were informed by their practice, had seen visible evidence on-site such as a marquee or signage, or learnt through news reports or by word-of-mouth. Among those who were unaware, 27% indicated they would have felt reassured to contact general practice had they known that the patient groups were being kept apart.

Table 1 shows a sample of the perspectives of our respondents concerning their communication with general practice. Half (A-M) describe experiences of consultations or messaging about the changed delivery, the remainder (N-Z) indicate their preferences and suggestions for this. Examples have been selected to highlight themes evident in the surveys, rather than to represent, for example, satisfaction with this communication. As might be expected, these may be experienced positively or negatively, dependent upon individual circumstances:

**Table 1.** Selected perspectives of respondents on their communication with general practice, March to September 2020. Quotations were selected to demonstrate themes across responses rather than to represent levels of satisfaction.

ID	Experiences of communication	ID	Preferences and suggestions for communication
A	“Surprised at how happy I was with the phone/video appointments. Definitely better than waiting in the surgery for things that don't require face to face”	N	“The explanation could have been better described in a very simple graphic”
B	“Lots of my friends don’t have access to technology and I know this has caused problems for them. Simple things like ‘phone surgery when you arrive’, they don’t have a mobile phone!”	O	“I have been getting my info from the Facebook site of a very good practice - Alvanley in Manchester, over 200 miles away!” [ https://www.facebook.com/Alvanleyfamilypractice/ ]
C	“[Knowing about separation of COVID-19 patients] would have given me more confidence to look for help. I had not been in contact with people due to having diabetes and asthma and still don't wish to attend medical settings as I am unsure what the processes are”	P	“Simply making it clear that GP appointments were still available would help - the message on the online appointment system states they're not taking place”
D	“I don’t think patients with COVID-19 were seen by GPs...they were told to contact the hospital or [NHS] 111”	Q	“Via a leaflet through the door, or through the post. Some people don't have a computer or feel comfortable to use one”
E	“When phoning the practice, there’s a very long set of recorded messages about COVID before the piped music kicks in. It’s not unusual to be hanging on for 15 minutes without any indication of whether you’re in a queue”	R	“I think that phone calls, sending photographs and video links are not an acceptable alternative for face-to-face consultations … . I am happy for these things to continue if people want them but they shouldn't be assumed acceptable or suitable for everyone”
F	“I like the phone appointments. These have all been same day call backs which has been excellent”	S	“I think the surgery could have sent letters to all their patients explaining the changes in appointments”
G	“I was confused about whether I should have originally shielded or not. Positive info is better than assuming that as I have not received a letter all is well”	T	“I would find it helpful if there was an easy way of accessing the latest information without wading through lots of out-of-date material. Is there a way of signposting this more readily?”
H	“I find having to queue outside to speak to a receptionist who has no access to her computer … I don’t want to talk in front of a queue in the car park”	U	“I think some posters outside the health centres locally could have helped as phone lines got really busy”
I	“My anxiety has increased since lockdown as I feel uncomfortable and a little incompetent with the current situation when contacting primary care services”	V	“Phone was fine for me, but I guess information on local radio, TV and newspapers also helpful to some people”
J	“Video calls via my mobile were not effective”	W	“My GP has texted us throughout which is the best option in my opinion”
K	“I knew I wouldn’t be able to talk to a GP who knows me and I thought, because of the crisis, I should manage on my own”	X	“I think everyone should have been sent a text or email message. If they did not have access to either they should have had a phone call. It was very difficult to find the information and difficult to know what to do at the surgery”
L	“Most of the information provided on the GP website and in their recorded messages is general (ie, government advice, NHS advice) and not specific to local circumstances. Because phoning the practice was impossible, there was a tendency to make assumptions about what this means”	Y	“I welcome more use of e-consult and would like more use of video consult rather than phone call. More details of when GP phone calls were to arrive would be good; DPD delivery can tell me when they will arrive; how about a similar system for GP patient videos, update continuously via an app”
M	“Initially unsuccessful as I booked phone appointment for 30 min slot between dropping kids off and starting work and GP didn't call until after 30 mins. Eventually rearranged. Got a face to face appointment soon after”	Z	“Patients who cannot access the telephone independently should still be offered face to face appointments. I feel [X,Y,Z’s] human rights are being compromised by having to talk through me because they cannot use the phone, [they] wear hearing aids and [X] has Alzheimer’s and gets confused on the phone”

*Access to healthcare*

While respondents A,F,W and Y benefitted from the use of digital technology and remote consultations, respondents B,J,M,Q and R saw potential barriers to accessing healthcare in this way. Busy telephone lines and unclear answerphone messages were also common issues (E,L,U).

*Person-centred care*

Some patients preferred the modified forms of delivery, finding them more convenient (A,F,Y); for others, choice (H,Q,R), privacy (H,Z), dignity (Z) and continuity of care (K) could be compromised.

*Messaging*

Some respondents reported receiving sufficient, regular or timely communication from their practice (F,W) and 27 identified or described the use of hubs or zoned practices locally to them (data not shown). For others however, confusion arising from unclear, out-of-date or insufficient messaging was evident (C,D,G,I,L,P,T,X) and this could cause anxiety (C,I). Suggestions and preferences for explaining the relevant changes included the use of social media (O), graphics and posters (N,U), local broadcasting and newspaper coverage (V), and sending letters to patients (S). It was apparent that clear, relevant information from respondents’ practices and then other local sources was preferred. National sources of information were seen as less useful.

## Discussion

### Healthcare seeking

Of the 105 respondents with symptoms in our surveys, half reported not seeking advice or delaying doing so, most commonly to reduce demand on healthcare services and to a lesser extent due to fear of COVID-19, concurring with national and international findings. ^6^^–^^8^ These reasons, together with those of perceived or actual access issues, and differing preferences for the altered consultation modes were also shared with an NHS survey of 6614 patients in South-West England [personal communication, Dr L. Farbus, NHS England and NHS Improvement, 22nd September 2020].

Despite promotion of the NHS 111 service during the pandemic, only 36% of respondents either called 111 or accessed the NHS 111 website, with a mere 5% using this service alone. This may partly be due to the misunderstanding by some that only those with COVID-19 symptoms were to use the service. However, it was also clear that respondents wanted local, relevant communication, preferably from their own practices. Indeed 84% of people contacted their own practice directly, with 40% using no other method.

### Satisfaction with contacts

Satisfaction levels among respondents who sought advice in general practice remained high at 78%, similar to pre-pandemic levels. ^9^ Among those receiving face-to-face consultations, satisfaction was 100%. 20 respondents were dissatisfied however. Half of these were unable to make contact, while others were unhappy with the outcomes of contacts made, typically related to the availability and modes of consultations.

### Changed models of delivery

Our surveys indicate that the changes to service delivery have decreased equity of access. While some respondents benefitted from video and telephone consultation availability, for example where it could be hard to fit face-to-face appointments around caring or work responsibilities, others experienced reduced access due to lack of relevant information, fear, loss of choice, logistic and/or technological barriers. The necessary speed of change has undoubtedly impacted all parties and limited co-production of the new models with patients and staff. It is of interest however that a small number of individuals successfully self-managed conditions they would previously have brought to general practice, including self-monitoring of blood pressure and treatment of corns.

### Communication of changes

While some people were well-informed about the changes to face-to-face consultations, public awareness was generally low, and some respondents indicated that better understanding would have reassured them to seek healthcare advice. Some ambiguity in messaging was apparent, with both the understanding that COVID-19 patients were not being seen in general practice and, contrastingly, that patients without COVID-19 were not being seen, indicated in our survey. Email communication in January 2021 with a small number of respondents suggested that both the avoidance of general practice and reasons for this were still present, with some people remaining unable to make contact and/or having received minimal communication from their practice. Conversely, regular communication was reported by some respondents. This may be contributing to the different messages coming from the public, media and general practice concerning the availability of general practice appointments. ^10^^,^^11^

The differences identified in both the communication received and its comprehension are perhaps unsurprising, given that national focus has been on secondary care of people with COVID-19, ^12^ focus in general practice has necessarily been on adapting delivery and providing safer care, ^13^ and that patients have been faced with volumes of information from multiple sources throughout the pandemic. ^14^^–^^16^ It is clear though, that this has impacted on patient experience of general practice, causing confusion and increased anxiety in some, while delivering improved access for others. NHS guidance indicates the importance of informing the public of changes, and the need for accessible patient communication has also been identified. ^1, 10, 15, 17, 19^ Evidence of regular communication by individual GP practices with their patients is available ( https://www.facebook.com/Alvanleyfamilypractice/; https://youtu.be/kEXOSl0cIaA) ^18^ and it is likely that the COVID-19 vaccination campaign has also re-established connection with some patients. Clear, current and specific messaging detailing the local measures in place to keep people safe, will empower others.

### Limitations

This online survey of 150 people was largely local to South-West England, an area of relatively low COVID-19 incidence to date. While our findings concerning the use of general practice during the pandemic reflect those obtained in other regional and national surveys, studies of populations in regions with different demographics and including those without internet access, may identify additional themes and establish whether our outcomes concerning knowledge of adapted general practice delivery are representative nationally.

## Conclusions

150 survey respondents have provided insights into the experience and communication of general practice between March and September 2020. While the adapted models of delivery were preferred by some patients, they were inaccessible to others. Possible reasons for general practice avoidance were also indicated, including a significant lack of awareness of the measures taken to optimise safety during face-to-face consultations. Evaluation of all delivery models, incorporating perspectives from both staff and patients, as well as the checking of current messaging, should help to ensure that all patients are able to access general practice.

## Data Availability

Figshare: Survey A responses, https://doi.org/10.6084/m9.figshare.14269967.v1 ^20^ Free text responses in surveys A and B cannot be made openly accessible as it is not possible to successfully anonymise each of these responses and the data cannot be shared outside of the research team. Either respondents to the surveys, their friends or family, healthcare sites and staff may be identifiable. Some anonymised responses reflective of overall responses are available in Table 1. Figshare: Survey A questions, https://doi.org/10.6084/m9.figshare.14269469.v1 ^21^ Figshare: Survey A flyer, https://doi.org/10.6084/m9.figshare.14269475.v1 ^22^ Figshare: Survey B questions, https://doi.org/10.6084/m9.figshare.14269478.v1 ^23^ Figshare: Reporting of the design, conduct and analysis of surveys A and B, https://doi.org/10.6084/m9.figshare.14269454.v1 ^24^ Data are available under the terms of the Creative Commons Attribution 4.0 International license (CC-BY 4.0).
