# Public perception of NHS general practice during the first six months of the COVID-19 pandemic in England

**DOI:** 10.12688/f1000research.52392.2

**Published:** 2021-11-01

**Authors:** Lorna J. Duncan, Kelly F.D. Cheng

**Affiliations:** 1 Centre for Academic Primary Care, Population Health Sciences, Bristol Medical School, University of Bristol, Bristol, UK; 2 Bristol Medical School, University of Bristol, Bristol, UK

**Keywords:** COVID-19, general practice, coronavirus, SARS-CoV-2 transmission, delivery model, face-to-face consultation, patient communication, patient experience

## Abstract

**Background: **In March 2020, the delivery of NHS general practice consultations was rapidly modified to mitigate the spread of COVID-19. Remote triage and consultations became the default, with adapted models for face-to-face contact if clinically required. This study aimed to gain insight into public perception of these adaptations.

**Methods: **Two online surveys were developed, and conducted in August and September 2020. Survey A, open to adults (>18 years) receiving the link to it, considered respondents’ perspectives on healthcare contacts since March 2020, and their understanding of the adapted delivery. Survey B, open to survey A respondents only, then considered how healthcare communication had been received and individual preferences for this. Survey participation was voluntary.

**Results: **The perceptions of 150 members of the public were obtained. 105 had considered contacting general practice,

although half avoided this or delayed doing so for longer than usual. While some patients did so ‘to help the NHS’, others experienced reduced access for reasons including concerns about telephone consultations and about COVID-19 safety. Some however reported benefitting from remote consultation availability and regular texts/emails from their practice.

68% (102/150) of respondents were unaware that patients with COVID-19 were seen separately from other patients during general practice appointments. 27% in survey B who had avoided or delayed contact said they would have felt more comfortable contacting general practice had they known this.

**Conclusions: **Experience and use of the adapted general practice models varied. Some patients felt their access to healthcare was reduced, often due to technological requirements. For some who found attending face-to-face appointments difficult however, remote contact was advantageous. Most of those surveyed were unaware of the COVID-19 control measures in place during face-to-face general practice consultations. Assessment of adapted delivery model accessibility and clearer public messaging about the changes may help reduce inequalities.

## Introduction

With the onset of the COVID-19 pandemic, delivery of general practice consultations changed rapidly and extensively throughout England as in other countries. ^1^ In March 2020, National Health Service (NHS) Standard Operating Procedure (SOP) was adapted to minimise cross-infection and remote triage and consultation became the default model. Face-to-face consultations were used only when clinically necessary ^2^ and their proportions dropped from 80% of total general practice consultations in February 2020, to 47% in April 2020, as shown in Table 1. [Table includes selected months only - from February to May 2020, and August 2021 - for illustrative purposes]. These proportions remained low in May and June 2020, then rose gradually from the end of the first national lockdown in July 2020. Nevertheless, face-to-face appointments remained considerably below pre-pandemic levels at 58% of totals in the most recently available, August 2021, data. ^3^

**Table 1:** Changes to English NHS general practice appointment modes and totals during the COVID-19 pandemic. ^3^

Consultation mode	February 2020 appointments		March 2020 * appointments		April 2020 appointments		May 2020 appointments			August 2021 appointments	
	millions	%	millions	%	millions	%	millions	%		millions	%
Face-to-face	19.229	80	15.921	67	7.480	47	7.730	47		13.711	58
Telephone **	3.322	14	6.637	28	7.651	48	7.814	48		9.128	38
Video/online	0.173	1	0.127	1	0.044	0	0.042	0		0.096	0
Home visits ***	0.227	1	0.173	1	0.101	1	0.112	1		0.152	1
Unknown mode	0.858	4	0.914	4	0.559	4	0.678	4		0.668	3
Total consultations	23.810	100	23.772	100	15.835	100	16.375	100		23.754	100

Selected months shown for illustrative purposes. Full data available from NHS Digital. ^3^

* NHS Standard Operating Procedures to mitigate COVID-19 were introduced during March 2020.

** Telephone consultations; telephone triage may also be included if logged as individual, rather than block, appointments.

*** Home visits included if logged as individual, rather than block, appointments.

The reduction in face-to-face appointments was offset by increased proportions of telephone contacts, which rose from 14% of totals in February 2020, to 48% two months later ( Table 1). They remained above pre-pandemic levels, at 38% in August 2021. Recorded numbers of video or online consultations and home visits meanwhile were consistently low, jointly comprising around 2% of totals during this period.

**Figure 1.**
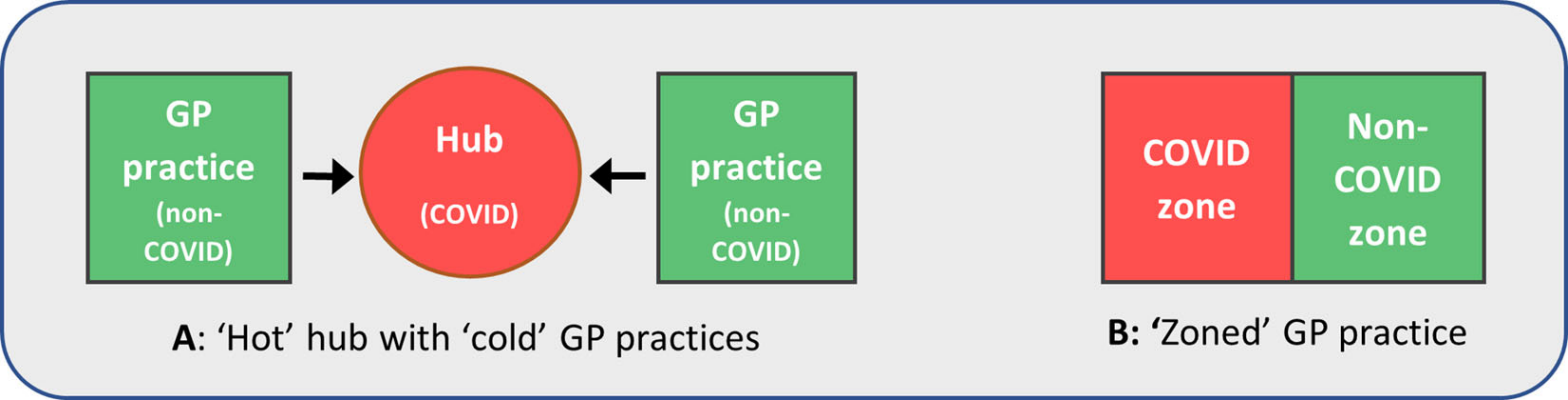
Typical models used to separate patients with suspected or diagnosed COVID-19 from others in general practice. Variations of these models could be used, as well as designated ‘COVID-19’ and ‘non-COVID-19’ home visiting services.

Besides being reduced in number during the pandemic, necessary face-to-face appointments required reorganisation in order to comply with Infection Prevention and Control (IPC) guidance. Patients with suspected or diagnosed COVID-19 (‘COVID-19 patients’) were to be seen separately from other patients. ^2^ Our June 2020 study reported on the locally adapted models used to deliver NHS face-to-face general practice consultations during the first national lockdown in England. ^4^ While several nuances to the models were apparent, the two most typical are shown in Figure 1. In model A, COVID-19 patients were seen at a ‘hot’ hub - a site shared between several locally collaborating practices. All other patients were seen at ‘cold’ GP practices. In model B meanwhile, COVID-19 and other patients were seen at their own practice, but in two separate ‘zones’. These were carefully managed to minimise cross-contamination, with staff working in one zone only, and separate entrances and exits.

Management of both face-to-face and remote general practice delivery during the pandemic has been evaluated, incorporating the perspectives of healthcare professionals, in several countries. ^1^^,^^5^^-^^9^ The rapid switch from largely face-to-face to mainly telephone consultations was common and, while considered necessary, concern was raised about the consequent potential for increased clinical risk, decreased continuity of care and impact on communication with patients. This was exacerbated in some countries by the fact that, besides the changes in appointment modes, consultation numbers also dropped overall. ^1^ This is also shown in England in Table 1, with appointment totals dropping by around one-third between February and April 2020, ^3^ from almost 24 million to nearly 16 million per month respectively [in 2019, figures remained at around 23 million throughout these months]. Among possible reasons for this decrease were the change to total triage prior to arranging consultations, as well as public response to the rapidly adapted consultation models themselves. Indeed, some surveys identified avoidance of general practice by patients. ^1^^,^^10^^,^^11^ Some media reports however, suggested that at least some GP surgeries were either not open or could not be contacted, or that face-to-face appointments were not available, although this was refuted by organisations representing general practice. ^12^^,^^13^

The aims of this study were:

- to explore public experience and perceptions of general practice in the first six months of the pandemic (March-Sept 2020);
- to understand public awareness of the changes to general practice and the ways in which information was received about this.

## Methods

Online survey methodology was used to accommodate the COVID-19 restrictions in place in England in the late summer and autumn of 2020. Two surveys, A and B, were conducted sequentially to identify the public’s experience and perceptions of general practice in England from March to September 2020. Both those who had and had not needed to use general practice during this period were included.

### Survey design

Survey A considered:

• respondents' contacts with primary care for any symptoms (COVID-19 and/or non-COVID-19) since March 2020, their experience and satisfaction with this;
• respondents' awareness of the physical separation of COVID-19 patients from other patients during general practice face-to-face consultations.

Survey B then considered:

• how respondents who knew that COVID-19 patients were separated from other patients during consultations gained this knowledge;
• COVID-19 information sources used and preferred by respondents.

Survey questions were developed by the study team and made available through JISC online surveys. They were pre-tested on five people (two experienced in survey design and three lay representatives), and minor changes to wording were made for clarity. The final questionnaires, and flyer giving password access to survey A, are available as *Extended data.* ^14^^-^^16^

While NHS ethics approval was not required for this evaluation of general practice delivery, the study was designed and carried out according to UK and other guidance. ^17^^,^^18^ Steps were taken to maximise safety for respondents, as follows. Surveys were distributed to adults (aged 18 years or over) who had elected to receive Patient and Public Involvement and Engagement newsletters or emails, as well as staff at NHS England & NHS Improvement and at one unitary authority. All who completed the surveys did so voluntarily and in confidence. Security of the data they provided was optimised using the JISC online survey tool which is designed for academic use and is General Data Protection Regulation (GDPR) compliant and certified to ISO 27001 information security standards. The data was password-protected and accessible only by the study team. Minimal personal data was required from those surveyed (postcode or address, at town level only). Contact details (email address or telephone number) could be volunteered after the final survey question. A hard copy of responses – minus any contact details – was stored in a locked filing cabinet and kept for one year from closure of the study.

Information on page 1 explained the purpose of each survey and that it was likely to take less than 5 minutes to finish. Completion by both those who had and had not needed to contact their GP, was requested. A privacy statement on page 2 explained how data would be stored and anonymity maintained, including in any presentations, reports or papers; and that respondents could choose whether to provide their contact details. Informed consent, both to participation and to publication of anonymised results, was implied through survey completion. A study email address was also provided, for respondents to indicate their willingness to help further, to request study outputs, or for any other reason. Our report was sent to those who requested it, indicating that it was yet to be peer reviewed, with information provided on locating any updated versions. An invitation to provide feedback was given, including any suggestions for future study.

Survey questions considered whether respondents were aware that COVID-19 patients were seen separately from other patients during consultations; how they had sought and received communication about general practice during the pandemic, and their preferences for this. Experience of accessing general practice was also requested of those who had used, or tried to use, the service. 

### Data collection

Survey A was open to any adult receiving details of it, including the password. These details, with the flyer, were distributed via email by the Patient and Public Involvement and Engagement (PPIE) team at the Centre for Academic Primary Care, University of Bristol, to a list of their PPIE contacts. A newsflash was also placed in People in Health West of England’s (PHWE) newsletter. PHWE promotes public involvement in evidenced-based service improvement. Details were additionally distributed by Dr L. Farbus, Head of Stakeholder Engagement, Direct Commissioning NHS England and NHS Improvement South West, to other staff within the organisation, and by South Gloucestershire Council to their staff. Survey B was open to survey A respondents who agreed to help further via a link distributed from the survey website to email addresses supplied.

Survey A was open August 4 ^th^—September 9 ^th^ 2020; survey B between August 19 ^th^ and September 14 ^th^ 2020.

In January 2021, an email was also sent from the study email address to 11 survey B respondents who had indicated their willingness to help further. They were asked whether they were more or less worried about contacting their GP than previously and whether they had made contact and/or received any further communication about general practice.

### Data analysis

Descriptive statistics (counts and percentages) of closed questions were provided within the JISC online survey analysis tool. Free-text responses, generally no more than two sentences in length, were analysed numerically and/or narratively as appropriate.

Familiarity with the data was obtained by reviewing responses in two ways: first, as each respondent's individual answer set, and then as complete answer sets for each question. Topics of interest were then analysed using closed and/or open responses, as follows:

*Locations of respondents* [identified in survey A, question 13], were compared with locations served by Clinical Commissioning Groups (organisations responsible for planning and commissioning community NHS services in England), using their websites (from links at NHS England). Links were made accordingly and counts and percentages of respondents in each CCG were calculated.

*Respondent journeys through healthcare* were followed using answers to questions 1-11 in survey A. A flowchart was created summarising the stages at which respondents ‘left’ the journey through healthcare to a consultation (those who had no symptoms left at stage 1 for example; those who had symptoms, but did not seek help, left at stage 2, etc). Counts of those leaving at each stage were made, in addition to numbers of those reporting COVID-19 symptoms, type of help-seeking used and satisfaction with this.

*Awareness of the separation of patients with COVID-19 symptoms from other patients during consultations* [survey A, question 14] - counts and percentages were obtained within the survey tool. Of the respondents who reported knowing that COVID-19 patients were seen separately to those with non-COVID-19 symptoms, cross-referencing was used to question 12 in survey A and question 1 in survey B to identify those who worked in general practice and other ways in which they had indicated gaining this knowledge. For those who indicated they were unaware, question 1 in survey B was used to establish whether they felt this knowledge would have altered their decision-making around advice seeking.

*Communication methods used and preferred* to obtain information about general practice locally and about COVID-19 generally [survey B, questions 2 and 3]. A wide variety of responses were received, and respondents gave one or several communication methods and suggestions in their answers. Due to the relatively small respondent number in survey B, numeric analysis was not appropriate. Rather, the responses for each question were grouped into themes where possible. Typical quotations were then selected to represent these themes, together with other, less common ideas with the aim that all were represented.

Additional reporting of methodology, following guidance for online surveys, ^18^ is available as *Extended data.* ^19^

## Results

### Survey A respondents

**Figure 2.**
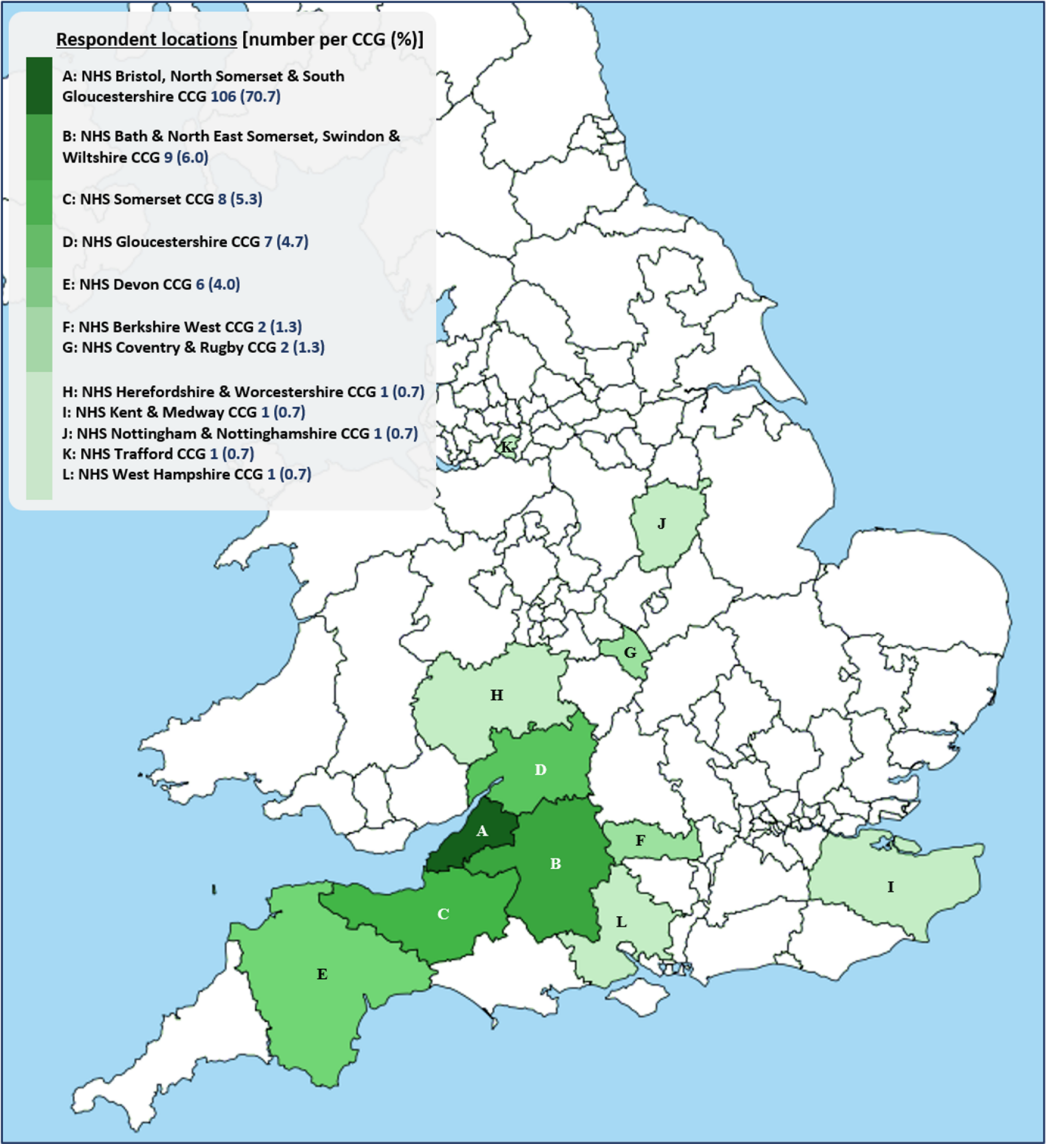
Locations of survey A respondents (n=150) by Clinical Commissioning Group (CCG). 5 respondent locations unknown.

A total of 150 people completed survey A. Although they were not required to disclose personal details, ages of respondents who volunteered such information ranged from 27 to 71, and both women and men were included. People who had not needed to use general practice during the surveyed period were represented, as were those with new and/or pre-existing conditions. Figure 2 shows respondent locations in relation to the Clinical Commissioning Groups (CCGs) planning and commissioning NHS services in their areas. Our respondents lived within the boundaries of 12 CCGs, labelled A-L on the map. 91% lived in South-West England (CCGs A-E), a region of relatively low COVID-19 incidence throughout 2020. ^20^ 71% were from NHS Bristol, North Somerset and South Gloucestershire CCG. 15 respondents were healthcare professionals, with three working in general practice. Closed responses to survey A questions are available as *Underlying data.* ^21^

*Decision to contact general practice or NHS 111*

**Figure 3.**
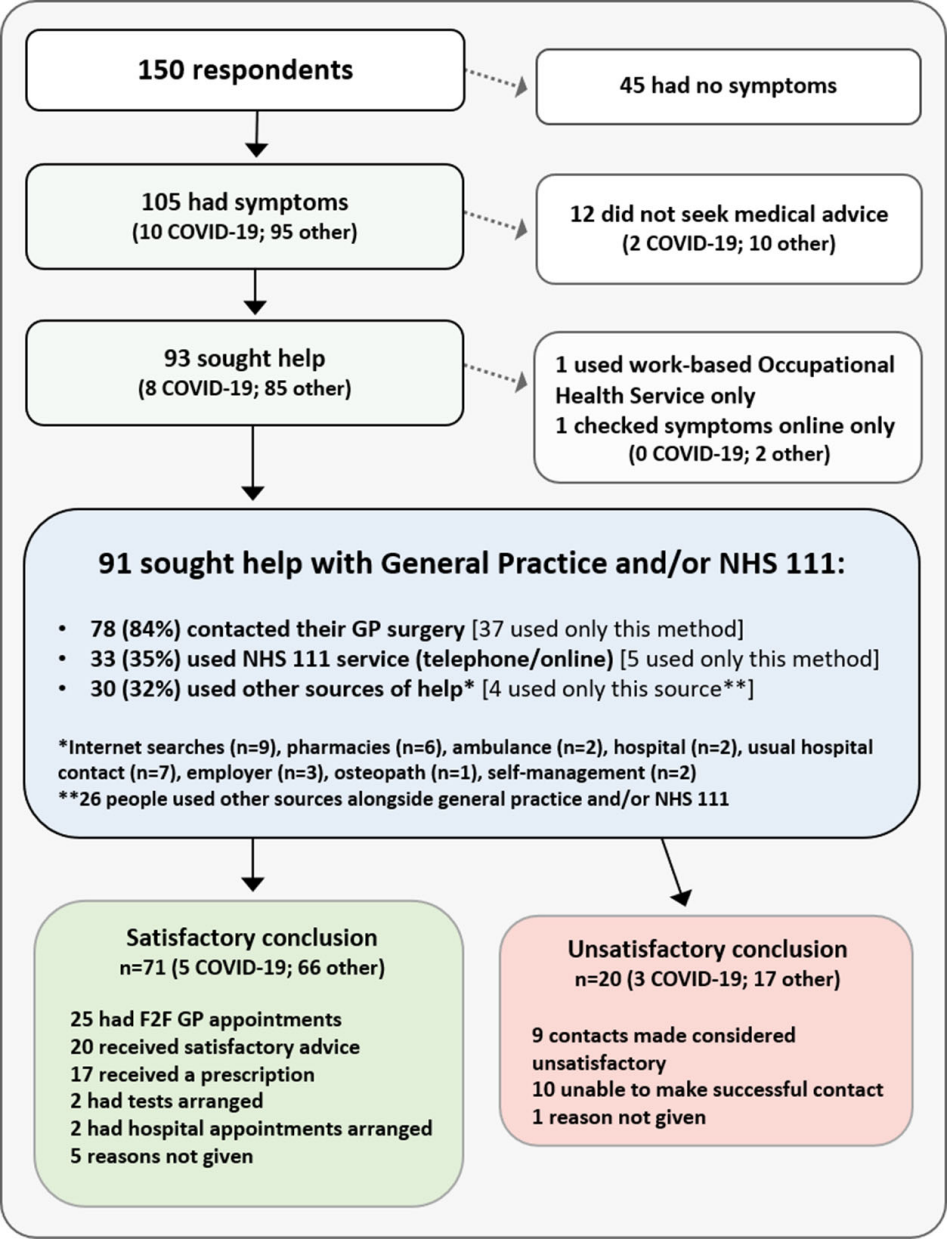
Survey A respondents’ use of healthcare, March-Sept 2020. NHS 111 is a national telephone helpline and website for patients.

In total, 70% (105/150) of survey A respondents reported having considered contacting general practice or NHS 111 (a national telephone helpline and website) since March 2020; 10 thought they may have had COVID-19. Figure 3 shows the healthcare interactions of all respondents between March and September. It can be seen that twelve symptomatic respondents did not seek advice; a further 41 reported delaying doing so for longer than usual (data not shown). Excepting four people who managed their own symptoms, these two groups represented 47% of symptomatic respondents. The most common reason for this, given by 39% of these respondents, was a desire to ‘help the NHS’. Other factors included access issues (either anticipated or experienced), lack of face-to-face consultations or feeling uncomfortable with telephone consultations, and fear of contracting COVID-19.

By contrast, 11% of symptomatic respondents reported contacting general practice more quickly than usual, mainly due to symptom severity or anxiety. In two cases however, patients required prompt advice to establish whether they should self-isolate, according to COVID-19 regulations.

*Contacts made*

Irrespective of time taken to seek help, a total of 93 people - 89% of those who were symptomatic - did so. While two people used occupational health or online searches only, the remaining 91 used NHS healthcare, as detailed in Figure 3. The vast majority - 78 people - contacted their GP surgery, 37 using only this method. 33 respondents used the NHS 111 service (15 by telephone, 18 accessing it online), but only 5 used it alone. Several other sources (identified in Figure 3) were also used by 30 people, but most used these alongside general practice or NHS 111 contacts.

*Satisfaction with contacts*

As Figure 3 illustrates, 78% (71/91) of respondents who contacted general practice and/or NHS 111 felt they received the help they needed. This included all 25 who had face-to-face appointments, despite one-fifth of these having had initial concerns about attending related to COVID-19 safety. 20 respondents however were dissatisfied, with reasons involving the inability to make successful contact, or unsatisfactory outcomes where contact was made, due mainly to the unavailability of appointments and dissatisfaction with remote appointments.

*Understanding of adapted delivery models*

The evident reluctance to seek help by almost half of symptomatic patients in this survey was explored by investigating all 150 respondents’ understanding of the changed general practice delivery models. A large proportion (68%) reported not knowing whether COVID-19 patients and non-COVID-19 patients were isolated from each other during face-to-face consultations. Similar proportions were seen in each sub-group in Figure 3. Further confusion was indicated by the fact that 25% of those who sought help (23/93) were unaware that face-to-face general practice consultations could happen at all during lockdown.

### Survey B respondents

Survey B was distributed to 71 survey A respondents who indicated their willingness to help further, and 56 of these (79%) completed it. Their characteristics were checked using responses to the first survey and this sub-group was found to be similar in terms of location (96% lived in South-West England compared to 91% in survey A) and occurrence of symptoms (73% compared to 70%). However, awareness that COVID-19 patients were kept physically apart from patients with non-COVID symptoms was somewhat higher in these respondents, at 39% compared to 32% in survey A respondents.

*Communication regarding COVID-19 and changes to general practice service delivery*

In total, 28% of respondents who knew about the measures used to control COVID-19 during face-to-face consultations had working links to general practice (as GP staff, members of GP Patient Participation Groups, or by providing delivery services to general practice). Others had been informed by their practice, seen visible evidence on-site such as a marquee or signage, or learnt through news reports or by word-of-mouth. Among those who were unaware, 27% indicated they would have felt reassured to contact general practice had they known that the patient groups were being kept apart.

Table 2 shows a sample of the perspectives of respondents to our surveys concerning their communication with general practice. Each quotation has been allocated an identifier (A-Z) to enable reference to it in the remainder of this section. Half (A-M) describe experiences accessing the adapted delivery models, or of messaging about the changes; the remainder (N-Z) indicate respondents’ preferences and suggestions for this. Examples have been selected to highlight the breadth of themes evident in the survey, and do not represent the frequency with which they arose, or satisfaction with this communication (which is shown in Figure 3). As might be expected, communication methods can be seen to be experienced positively or negatively, dependent upon individual circumstances and preferences.

**Table 2.** Selected perspectives of respondents on their communication with general practice, March to September 2020. Quotations were selected to demonstrate themes across responses rather than to represent levels of satisfaction. *Each quotation has been assigned an identifier (A-Z) for referencing within the text.

ID*	Experiences of communication	ID*	Preferences and suggestions for communication
A	“Surprised at how happy I was with the phone/video appointments. Definitely better than waiting in the surgery for things that don't require face to face”	N	“The explanation could have been better described in a very simple graphic”
B	“Lots of my friends don’t have access to technology and I know this has caused problems for them. Simple things like ‘phone surgery when you arrive’, they don’t have a mobile phone!”	O	“I have been getting my info from the Facebook site of a very good practice - Alvanley in Manchester, over 200 miles away!” [ https://www.facebook.com/Alvanleyfamilypractice/ ]
C	“[Knowing COVID-19 patients were isolated from others] would have given me more confidence to look for help. I had not been in contact with people due to having diabetes and asthma and still don't wish to attend medical settings as I am unsure what the processes are”	P	“Simply making it clear that GP appointments were still available would help - the message on the online appointment system states they're not taking place”
D	“I don’t think patients with COVID-19 were seen by GPs...they were told to contact the hospital or [NHS] 111”	Q	“Via a leaflet through the door, or through the post. Some people don't have a computer or feel comfortable to use one”
E	“When phoning the practice, there’s a very long set of recorded messages about COVID before the piped music kicks in. It’s not unusual to be hanging on for 15 minutes without any indication of whether you’re in a queue”	R	“I think that phone calls, sending photographs and video links are not an acceptable alternative for face-to-face consultations … . I am happy for these things to continue if people want them but they shouldn't be assumed acceptable or suitable for everyone”
F	“I like the phone appointments. These have all been same day call backs which has been excellent”	S	“I think the surgery could have sent letters to all their patients explaining the changes in appointments”
G	“I was confused about whether I should have originally shielded or not. Positive info is better than assuming that as I have not received a letter all is well”	T	“I would find it helpful if there was an easy way of accessing the latest information without wading through lots of out-of-date material. Is there a way of signposting this more readily?”
H	“I find having to queue outside to speak to a receptionist who has no access to her computer … I don’t want to talk in front of a queue in the car park”	U	“I think some posters outside the health centres locally could have helped as phone lines got really busy”
I	“My anxiety has increased since lockdown as I feel uncomfortable and a little incompetent with the current situation when contacting primary care services”	V	“Phone was fine for me, but I guess information on local radio, TV and newspapers also helpful to some people”
J	“Video calls via my mobile were not effective”	W	“My GP has texted us throughout which is the best option in my opinion”
K	“I knew I wouldn’t be able to talk to a GP who knows me and I thought, because of the crisis, I should manage on my own”	X	“I think everyone should have been sent a text or email message. If they did not have access to either they should have had a phone call. It was very difficult to find the information and difficult to know what to do at the surgery”
L	“Most of the information provided on the GP website and in their recorded messages is general (ie, government advice, NHS advice) and not specific to local circumstances. Because phoning the practice was impossible, there was a tendency to make assumptions about what this means”	Y	“I welcome more use of e-consult and would like more use of video consult rather than phone call. More details of when GP phone calls were to arrive would be good; DPD delivery can tell me when they will arrive; how about a similar system for GP patient videos, update continuously via an app”
M	“Initially unsuccessful as I booked phone appointment for 30 min slot between dropping kids off and starting work and GP didn't call until after 30 mins. Eventually rearranged. Got a face to face appointment soon after”	Z	“Patients who cannot access the telephone independently should still be offered face to face appointments. I feel [X,Y,Z’s] human rights are being compromised by having to talk through me because they cannot use the phone, [they] wear hearing aids and [X] has Alzheimer’s and gets confused on the phone”

Quotations have been selected largely from survey B (n = 22), with 4 taken from narrative responses to survey A questions (quotes D,J,K and M). Both surveys were used to enable greater inclusion of respondent perspectives in the table.

*Access to healthcare*

While some respondents (represented by quotations A,F,W and Y) benefitted from the use of digital technology and remote consultations, others (illustrated by quotations B,J,M,Q and R) saw potential barriers to accessing healthcare in this way. Busy telephone lines and unclear answerphone messages were also common issues (E,L,U).

*Person-centred care*

Some respondents preferred the modified forms of delivery, finding them more convenient (shown in quotations A,F,Y); for others however, choice (H,Q,R), privacy (H,Z), dignity (Z) and continuity of care (K) could be compromised.

*Messaging*

Some respondents reported receiving sufficient, regular or timely communication from their practice (represented by quotations F and W) and 27 identified or described the use of hubs or zoned practices locally to them (data not shown). For others however, confusion arising from unclear, out-of-date or insufficient messaging was evident (C,D,G,I,L,P,T,X) and this could cause anxiety (C,I). Suggestions and preferences for explaining the relevant changes included the use of social media (O), graphics and posters (N,U), local broadcasting and newspaper coverage (V), and sending letters to patients (S). It was apparent that clear, relevant information from respondents’ practices and then other local sources was preferred. National sources of information were seen as less useful.

Email communication in January 2021 with a small number of respondents suggested that both the avoidance of general practice and the reasons for this were still present for some people, who remained unable to make contact and/or had received minimal communication from their practice. Conversely, regular communication was reported by others.

## Discussion

### Healthcare seeking

Of the 105 respondents with symptoms in our surveys, half reported not seeking advice or delaying doing so, most commonly to reduce demand on healthcare services and to a lesser extent due to fear of COVID-19, concurring with national and international findings. ^1^^,^^10^^,^^11^ These reasons, together with those of perceived or actual access issues, and differing preferences for the altered consultation modes were also shared with an NHS survey of 6614 patients in South-West England [personal communication, Dr L. Farbus, NHS England and NHS Improvement, 22nd September 2020].

Despite promotion of the NHS 111 service, only 36% of respondents either called 111 or accessed the NHS 111 website, with a mere 5% using this service alone. This may partly be due to the misunderstanding by some that only those with COVID-19 symptoms were to use the service. However, it was also clear that respondents wanted local, relevant communication, preferably from their own practices. Indeed 84% of people contacted their own practice directly, with 40% using no other method.

### Satisfaction with contacts

Satisfaction levels among respondents who sought advice in general practice remained high at 78%, similar to pre-pandemic levels. ^22^ Among those receiving face-to-face consultations, satisfaction was 100%. 20 respondents were dissatisfied however. Half of these were unable to make contact, while others were unhappy with the outcomes of contacts made, typically related to the availability and modes of consultations. Practical issues relating to the use of telephone triage prior to arranging consultations, including difficulty making contact and/or arranging suitably timed call backs, have previously been identified in general practice in England. Varied patient perspectives on this, both positive and negative, were also identified. ^37^

### Changed models of delivery

Our surveys indicate that the changes to service delivery have decreased equity of access. While some respondents benefitted from video and telephone consultation availability, for example where it could be hard to fit face-to-face appointments around caring or work responsibilities, others experienced reduced access due to lack of relevant information, fear, loss of choice, logistic and/or technological barriers. Similar themes have also been identified in national and international surveys of patients. ^23^^,^^24^ Furthermore, concern over the reduced availability of face-to-face appointments and impacts on patient-centred care, as well as on clinical risk, is shared by many GPs. ^25^^-^^27^ The necessary speed of change, together with general practice staffing issues, has undoubtedly impacted all parties and limited co-production of the new models. ^27^ It is of interest however that, during the pandemic, a small number of individuals successfully managed conditions they would previously have brought to general practice, including self-monitoring of blood pressure and treatment of corns.

### Communication of changes

While some people were well-informed about the changes to face-to-face consultations, public awareness was generally low, and some respondents indicated that better understanding would have reassured them to seek healthcare advice. Some ambiguity in messaging was apparent, with both the understanding that COVID-19 patients were not being seen in general practice and, contrastingly, that patients without COVID-19 were not being seen, indicated by different respondents. The apparent differences in success in contacting GPs, together with variability reported in the quantity and usefulness of communication received from practices, may have contributed to the different messages coming from the public, media and general practice concerning the availability of general practice appointments. ^12^^,^^28^

The differences identified in both the communication received and its comprehension are perhaps unsurprising, given that national focus has been on secondary care of people with COVID-19, ^29^ focus in general practice has necessarily been on adapting delivery and providing safer care, ^30^ and that patients have been faced with large amounts of information from multiple sources throughout the pandemic. ^31^^–^^33^ It is clear though, that this has impacted on patient experience of general practice, causing confusion and increased anxiety in some, while delivering improved access for others. NHS guidance indicates the importance of informing the public of changes, and the need for accessible patient communication has also been highlighted. ^2, 28, 32, 34, 35^ Evidence of clear, regular communication by individual GP practices with their patients is available ( https://www.facebook.com/Alvanleyfamilypractice/; https://youtu.be/kEXOSl0cIaA) ^36^ and it is likely that the COVID-19 vaccination campaign has also re-established connection with many patients. Indeed, total appointments in England were similar to pre-pandemic levels in the most recently available data (August 2021, Table 1). Clear, current and specific messaging, in different formats for optimum accessibility and detailing the local measures in place to keep people safe, will nevertheless help to empower those who remain hesitant. It should also facilitate appropriate IPC behaviours during face-to-face consultation and optimise outcomes of any interactions for both patients and staff.

### Strengths and limitations

A strength of this study was that understanding of our respondents' experiences of general practice during the pandemic was assisted by considering the communication they had received about this, and their awareness of the IPC procedures in place. In addition, we obtained the perspectives of those who had not needed to access general practice during the pandemic, as well as those who had (some of whom had accessed remote or face-to-face consultations, while others had avoided making contact). With the need to use general practice often unpredictable, it is important to ensure communication about changed processes is available to everyone, to enable both initial access to, and successful navigation through, the healthcare system.

Limitations to our survey include its small sample size and that it was largely local to South-West England, a region of relatively low COVID-19 incidence throughout 2020. We were also unable to include people without internet access, although some respondents described the reduced ability to obtain information and/or access healthcare of those they knew without computers or smartphones. Each of the above factors limits the generalisability of our study. However, our findings concerning the use of general practice during the pandemic reflect those obtained in other regional, national and international surveys. In relation to general practice communication, and to respondents’ understanding of the safer practices adopted during the pandemic, it is possible that surveys in regions with different demographics and including people without internet access, will identify additional themes and establish whether our outcomes concerning knowledge of adapted general practice delivery are found nationally.

## Conclusions

150 survey respondents have provided insights into the experience of, and communication about, general practice in England between March and September 2020. While the adapted models of delivery were preferred by some patients, they were inaccessible to others. Possible reasons for general practice avoidance were also indicated, including a significant lack of awareness of the measures taken to optimise safety during face-to-face consultations. Evaluation of all delivery models, incorporating perspectives from both staff and patients, as well as the checking of current messaging, should help to ensure that all patients are able to access general practice.

## Data availability

### Underlying data

Figshare: Survey A responses, https://doi.org/10.6084/m9.figshare.14269967.v1. ^21^

Free text responses in surveys A and B cannot be made openly accessible as it is not possible to successfully anonymise each of these responses and the data cannot be shared outside of the research team. Either respondents to the surveys, their friends or family, healthcare sites and staff may be identifiable. Some anonymised responses reflective of overall responses are available in Table 2.

### Extended data

Figshare: Survey A questions, https://doi.org/10.6084/m9.figshare.14269469.v1 ^14^

Figshare: Survey A flyer, https://doi.org/10.6084/m9.figshare.14269475.v1 ^15^

Figshare: Survey B questions, https://doi.org/10.6084/m9.figshare.14269478.v1 ^16^

Figshare: Reporting of the design, conduct and analysis of surveys A and B, https://doi.org/10.6084/m9.figshare.14269454.v1 ^19^

Data are available under the terms of the Creative Commons Attribution 4.0 International license (CC-BY 4.0).
