# Supplementary material for: Public perception of NHS general practice during the first six months of the COVID-19 pandemic in England
Source: F1000Res. 2021 Nov 1;10:279. Originally published 2021 Apr 8. [Version 2] doi: 10.12688/f1000research.52392.2 (PMC8749896; doi:10.12688/f1000research.52392.2)
Supplement: Peer review history

# Peer review: Reviewer response for version 2

**DOI:** 10.5256/f1000research.77948.r98362

**Published:** 2022-01-10

**Authors:** Karen McBride-Henry

**Affiliations:** 1 School of Nursing, Midwifery, and Health Practice, Victoria University of Wellington, Wellington, New Zealand

Thank you for the opportunity to review your article. I appreciate the care and thoughtfulness with which you have attended to my review. I am happy with the revisions to your paper and wish you well in your future research endeavours.

Is the work clearly and accurately presented and does it cite the current literature?

Partly

If applicable, is the statistical analysis and its interpretation appropriate?

Yes

Are all the source data underlying the results available to ensure full reproducibility?

Partly

Is the study design appropriate and is the work technically sound?

Yes

Are the conclusions drawn adequately supported by the results?

Yes

Are sufficient details of methods and analysis provided to allow replication by others?

No

Reviewer Expertise:

Primary health care, mixed methods research, peoples experiences of health care and health care access

I confirm that I have read this submission and believe that I have an appropriate level of expertise to confirm that it is of an acceptable scientific standard.

# Peer review: Reviewer response for version 2

**DOI:** 10.5256/f1000research.77948.r98361

**Published:** 2021-11-01

**Authors:** Joanne Parsons

**Affiliations:** 1 Warwick Medical School, University of Warwick, Coventry, UK

Thank you for the responses to the comments. These have all been addressed thoroughly and have resolved any of the points I had.

Is the work clearly and accurately presented and does it cite the current literature?

Yes

If applicable, is the statistical analysis and its interpretation appropriate?

Yes

Are all the source data underlying the results available to ensure full reproducibility?

Yes

Is the study design appropriate and is the work technically sound?

Yes

Are the conclusions drawn adequately supported by the results?

Yes

Are sufficient details of methods and analysis provided to allow replication by others?

Yes

Reviewer Expertise:

Primary care, vaccination adherence, remote consultations, digital healthcare interventions.

I confirm that I have read this submission and believe that I have an appropriate level of expertise to confirm that it is of an acceptable scientific standard.

# Peer review: Reviewer response for version 1

**DOI:** 10.5256/f1000research.55666.r93309

**Published:** 2021-09-07

**Authors:** Karen McBride-Henry

**Affiliations:** 1 School of Nursing, Midwifery, and Health Practice, Victoria University of Wellington, Wellington, New Zealand

Thank you for the opportunity to review the article; it was interesting to read and is timely given the current COVID-19 pandemic. Research that seeks to understand how people experience accessing health care is essential to deliver care effectively. The results have some similarities to findings from research that we have conducted here in New Zealand.

 I offer the following comments that will help to refine the article further.

1. The introduction literature was limited, citing only the authors’ research. I would encourage the authors to broaden out the introduction to include additional research.
2. Information provided on the study methods was limited. For example, how were the survey respondents identified? How was the survey distributed? Although this information is provided in the “Checklist for reporting results of internet surveys” for this study on Figshare, additional information related to the study methods should be in the article. No information on the analysis of the open text answers is available; this information needs to be presented in the article.
3. A significant issue with the research was the lack of ethical approval for the study. Who deemed the survey as ‘low risk’? If it was the authors, this raises concerns. The lack of detail around ethical practices embedded in the study is a weakness. For example, how was anonymity assured? What were the procedures embedded in this study that kept respondents ‘safe’?
4. The figures helped explain the survey results.
5. Referring to respondents by alphabet letters was somewhat confusing given the limited information about the method of analysis. It is not clear whether the alphabet letters referred to respondents from survey A or B. This information should be noted in Table 1, as it indicates potential bias in the survey respondents.
6. New findings are presented in the Discussion, in the ‘Communication of changes’ section; these should be removed.
7. It is also reported that the researchers were in email contact with the respondents; while this is not necessarily unusual, it raises additional concerns about how respondent information was managed given the lack of ethical approval or ethical information related to this research.
8. The study authors used descriptive statistics only, which given the small sample size, is appropriate but impacts the generalisability of the results; this needs to be more clearly articulated in the limitation section.

Thank you again for the opportunity to review this research.

Is the work clearly and accurately presented and does it cite the current literature?

Partly

If applicable, is the statistical analysis and its interpretation appropriate?

Yes

Are all the source data underlying the results available to ensure full reproducibility?

Partly

Is the study design appropriate and is the work technically sound?

Yes

Are the conclusions drawn adequately supported by the results?

Yes

Are sufficient details of methods and analysis provided to allow replication by others?

No

Reviewer Expertise:

Primary health care, mixed methods research, peoples experiences of health care and health care access

I confirm that I have read this submission and believe that I have an appropriate level of expertise to confirm that it is of an acceptable scientific standard, however I have significant reservations, as outlined above.

# Response

**Published:** 2021-10-29

**Authors:** Lorna Duncan

**Affiliations:** University of Bristol, UK

Dear Professor McBride-Henry,

 Many thanks for taking the time to review our paper and for your helpful comments. We have used these to improve our manuscript as indicated below in our responses to each of your points:

 1. *The introduction literature was limited, citing only the authors’ research. I would encourage the authors to broaden out the introduction to include additional research.*

 Many thanks for highlighting this, we agree and have now expanded our introduction. We prepared the manuscript as a brief report and, as you mention, linked it only to our related publication identifying the pandemic-related modifications made to English face-to-face GP consultations. We can see that the additions we have now made, incorporating both national and international consideration of general practice adaptations and clarification of the purpose of our evaluation, have strengthened the paper. A table has also been added which we hope more clearly demonstrates the changes to general practice consultation modes described in the text.

 We were interested to read your findings around patient experience and preferences for telehealth in general practice during the March-May 2020 lockdown in New Zealand. We noted the similarities in several of the themes identified in both our studies and have included your publication, as well as further relevant references, to provide greater perspective in our Discussion.

 2. *Information provided on the study methods was limited. For example, how were the survey respondents identified? How was the survey distributed? Although this information is provided in the “Checklist for reporting results of internet surveys” for this study on Figshare, additional information related to the study methods should be in the article. No information on the analysis of the open text answers is available; this information needs to be presented in the article.*

 We have now expanded on the *Survey design*, *Data collection* and *Data analysis *sections in the Methods, to address your points. We have incorporated data from the *“Checklist for reporting results of internet surveys” * on Figshare and supplemented this with additional information. As mentioned in point 1 above, we had prepared this manuscript as a ‘brief report’, referring to the checklist as supplemental information, but we agree with you that these additions have improved clarity in the report.

 3. *A significant issue with the research was the lack of ethical approval for the study. Who deemed the survey as ‘low risk’? If it was the authors, this raises concerns. The lack of detail around ethical practices embedded in the study is a weakness. For example, how was anonymity assured? What were the procedures embedded in this study that kept respondents ‘safe’?*

 We agree that we had not indicated fully in the text the ways in which we acted to minimise harm to our respondents, although we had given some detail in the “Checklist for reporting results of internet surveys” on Figshare. We have now incorporated this detail into our Methodology section and added further explanation around the ethical considerations, including issues of anonymity and safety of respondents. The comment that the surveys were deemed ‘low risk’ was added by the editorial team during processing for publication. We should have requested that this be removed and have now done so - we are grateful to you for highlighting this. The work was undertaken as an evaluation of general practice delivery and we hope it is now clear in our expanded methodology section that we have considered the safety of our respondents throughout and taken appropriate steps to ensure security of their data and to preserve their anonymity. In fact, the first half of their postcode (which indicates their town or similar area) was the only identifiable element required from any respondent. Where respondents volunteered further detail we ensured this was treated and stored confidentially according to the methodology indicated. We also provided our study email address to which people could write additionally if they wanted to. A small number did so and we replied as the study team. Although the potential for harm for respondents in recollecting negative experiences was possible, this was minimised in our survey by not requesting specific details of these, focusing largely on service delivery and its communication, rather than on respondents’ health conditions or on the detail of any consultations they may have had. In addition, we feel that the brief nature of the surveys, with no consequence incurred by the respondents for non-completion (this was not in any way linked to their healthcare and the distributors of survey A had no knowledge of whether or not they had completed it), enabled us to maximise safety for each respondent. Quotations were also selected for our report to  encompass our respondents’ reflections overall while maintaining their individual anonymity.

 4. *The figures helped explain the survey results.*

 Thank you, we are pleased they are helpful.

 5. *Referring to respondents by alphabet letters was somewhat confusing given the limited information about the method of analysis. It is not clear whether the alphabet letters referred to respondents from survey A or B. This information should be noted in Table 1, as it indicates potential bias in the survey respondents.*

 Many thanks for highlighting possible confusion in our table. We have now included an explanation both in the table and within the text that the letters linked to each example are identifiers given purely to enable reference to them within the text. Our aim was not to suggest any hierarchy in our ‘coding’ system, so the alphabet was used rather than numbers.

 The quotations detailed in Table 2 indicate the breadth of experiences, views and ideas expressed by our participants, but not the frequency with which these ideas were expressed. They may have been indicated by one or several participants in our sample population, but have been included to represent possible factors for consideration in future consideration and planning of general practice delivery. We have now indicated which survey the quotations came from - most were from survey B, as this was where questions concerning respondents’ preferences and suggestions for communication were asked (none of the included quotes in the right-hand column were derived from survey A). In the left-hand column 4 of the 13 responses shown (D,J,K,M) were given in survey A, and this is not unexpected as both surveys considered respondents’ experience of general practice and all open text responses were scanned for examples for inclusion in  this table. Our selection was made to include as many ideas as possible, with the views of all 150 participants being equally important. We have also clarified this within the manuscript - in the *Data analysis* [‘Communication methods used and preferred’] section in the Methods, and in the *Communication regarding COVID-19 and changes to general practice service delivery* section in the Results.

 6. *New findings are presented in the Discussion, in the ‘Communication of changes’ section; these should be removed.*

 Thank you, we have now moved this item to the end of the *Results* section.

 7. *It is also reported that the researchers were in email contact with the respondents; while this is not necessarily unusual, it raises additional concerns about how respondent information was managed given the lack of ethical approval or ethical information related to this research.*

 We have now clarified the limited nature of this email contact, as well as the management of all respondent information, in the *Data collection* section in the Methods.

 8. *The study authors used descriptive statistics only, which given the small sample size, is appropriate but impacts the generalisability of the results; this needs to be more clearly articulated in the limitation section.*

 We agree and have now clarified this in the appropriate section, which has been reorganised into a ‘strengths and limitations’ section in the Discussion.

 Many thanks for each of the points you have raised. In addressing each, we feel this has very much helped us to refine our report.

# Peer review: Reviewer response for version 1

**DOI:** 10.5256/f1000research.55666.r84461

**Published:** 2021-05-06

**Authors:** Joanne Parsons

**Affiliations:** 1 Warwick Medical School, University of Warwick, Coventry, UK

This articles provides an examination of the perceptions of general practice patients of adaptations made to appointments as a result of the COVID-19 pandemic. Participants completed online surveys to explore use and perceptions of delivery and healthcare communication. 

 This is a very timely and relevant article, and is interesting in providing understanding about patients' experience and perceptions of this area. Background and rationale for the need for the project is clear, and methodology and findings are reported clearly and logically, making it an interesting and accessible paper to read. 

 I have read the paper in detail, and there are a few minor areas that some clarification on would improve the paper for readers. 

**Introduction:**

- Paragraph 1; you mention the percentage of face-to-face consultations before and during COVID-19. It would be interesting to also include the percentages of consultations that were delivered using other (remote) methods during the time that face-to-face consultations were reduced. 

**Methods:**

- When you detail what Survey A considered, you state 'respondents' contacts with primary care for any symptoms' - it would be helpful to state if this refers to COVID-19 symptoms only, or any symptoms of any health condition. Just to ensure readers are clear. 
- When you explain that five people pre-tested the survey questions (two were experienced in survey design), who were the other three? Was there input from lay/PPI representatives?

**Results:**

- It would be interesting for readers to have a brief summary of the participants that responded to each of the surveys. Before you discuss the location of respondents, could you include the mean age, and percentage of respondents that were male/female?

**Understanding of adapted delivery models:**

- There are a couple of occasions in the results section where you refer to participants not being aware that COVID-19 patients were 'separated from others in face-to-face consultations.' This wording is slightly unclear and perhaps rewording to make it clear that practices have different processes in place to ensure that patients with COVID-19 are seen in a different location/different part of the practice to other patients (you discuss it clearly in the introduction section).
- You refer to respondents having 'working links to general practice' (p7), could you please provide some examples of such links?

**Discussion:**

- When you cite the personal communication from Dr L. Farbus - should this be a numerical reference in line with the other references?
- The only other comment I have, is that you do not include any strengths or implications in the Discussion. This research is really interesting, and likely has real-world implications for ensuring that clear messages are provided to patients about when and how it is appropriate to contact the practice, and the availability of appointments during a situation such as COVID-19. It would be useful for readers to understand about how the findings of this research can improve patient experience.

Is the work clearly and accurately presented and does it cite the current literature?

Yes

If applicable, is the statistical analysis and its interpretation appropriate?

Yes

Are all the source data underlying the results available to ensure full reproducibility?

Yes

Is the study design appropriate and is the work technically sound?

Yes

Are the conclusions drawn adequately supported by the results?

Yes

Are sufficient details of methods and analysis provided to allow replication by others?

Yes

Reviewer Expertise:

Primary care, vaccination adherence, remote consultations, digital healthcare interventions.

I confirm that I have read this submission and believe that I have an appropriate level of expertise to confirm that it is of an acceptable scientific standard.

# Response

**Published:** 2021-10-29

**Authors:** Lorna Duncan

**Affiliations:** University of Bristol, UK

Dear Dr Parsons,

 Many thanks for reviewing our paper and for your specific comments which we found very helpful. We have now addressed each of your points to improve our manuscript as indicated below:

 1. *Paragraph 1; you mention the percentage of face-to-face consultations before and during COVID-19. It would be interesting to also include the percentages of consultations that were delivered using other (remote) methods during the time that face-to-face consultations were reduced.*

 Thank you, we have now included the full breakdown of consultations in a new table, and have expanded on this in the Introduction text. We agree this has added useful perspective and we have also taken the opportunity to update the figures to include those most recently available (August 2021).

 2. *When you detail what Survey A considered, you state 'respondents' contacts with primary* *care for any symptoms' - it would be helpful to state if this refers to COVID-19 symptoms only, or any symptoms of any health condition. Just to ensure readers are clear.* 

 Thank you for highlighting this, we have now indicated in the Methods section that contacts were for any symptoms, both COVID-19 and/ or those related to other conditions.

 3. *When you explain that five people pre-tested the survey questions (two were experienced in survey design), who were the other three? Was there input from lay/PPI representatives?*

 We have now expanded this sentence to clarify that the remaining three people were lay representatives.

 4. *It would be interesting for readers to have a brief summary of the participants that responded to each of the surveys. Before you discuss the location of respondents, could you include the mean age, and percentage of respondents that were male/female?*

 While the only required data from our respondents was their location (at town or similar level) and we are unable to give statistics on age and gender, it is possible to say from the information given by some, that the known ages of our participants ranged from 20’s to 70’s, and both men and women were included. We have now included this detail in our Results section, and have also indicated that people with both chronic and acute conditions were included, as well as those who had not had symptoms requiring them to contact general practice. While our survey size limited the generalisability of our findings and we focused rather on gathering the breadth of perceptions of our respondents, we feel that, this supplemental information has offered useful context in addition to that available in the quotations in Table 2. Many thanks for your suggestion.  

 5. *Understanding of adapted delivery models:*

*There are a couple of occasions in the results section where you refer to participants not being aware that COVID-19 patients were 'separated from others in face-to-face consultations.' This wording is slightly unclear and perhaps rewording to make it clear that practices have different processes in place to ensure that patients with COVID-19 are seen in a different location/different part of the practice to other patients (you discuss it clearly in the introduction section).*

 Thank you for your suggestion, we have now clarified this throughout accordingly.

 6. *You refer to respondents having 'working links to general practice' (p7), could you please provide some examples of such links?*

 We have now included elaboration that these people with worked in general practice or were members of patient participation groups or made deliveries to general practice.

 7. *When you cite the personal communication from Dr L. Farbus - should this be a numerical reference in line with the other references?*

 We appreciate your comment and agree that this is sometimes the case. Here, we have complied with the style required for personal communication by f1000research.

 8. *The only other comment I have, is that you do not include any strengths or implications in the Discussion. This research is really interesting, and likely has real-world implications for ensuring that clear messages are provided to patients about when and how it is appropriate to contact the practice, and the availability of appointments during a situation such as COVID-19. It would be useful for readers to understand about how the findings of this research can improve patient experience.*

 Many thanks for highlighting this and for your interest in our findings. We have now  replaced the ‘limitations’ section with a fuller ‘strengths and limitations’ section. In this, we now highlight the importance of considering the communication of changed practices, including the checking of access to, and understanding of, this communication, for change to be implemented successfully, and of maximum benefit for both patients and health care professionals.

 Thank you for the points you have raised, each has been really useful in helping us to improve this report.
